# Pharmacological Basis of CD101 Efficacy: Exposure Shape Matters

**DOI:** 10.1128/AAC.00758-17

**Published:** 2017-10-24

**Authors:** Elizabeth A. Lakota, Justin C. Bader, Voon Ong, Ken Bartizal, Lynn Miesel, David R. Andes, Sujata M. Bhavnani, Christopher M. Rubino, Paul G. Ambrose, Alexander J. Lepak

**Affiliations:** a Institute for Clinical Pharmacodynamics (ICPD), Schenectady, New York, USA; b Cidara Therapeutics, San Diego, California, USA; c Eurofins Panlabs, Ltd., St. Charles, Missouri, USA; d University of Wisconsin—Madison, Madison, Wisconsin, USA

**Keywords:** echinocandin, PK/PD, exposure shape, single dose, pharmacokinetics/pharmacodynamics

## Abstract

CD101 is a novel echinocandin with concentration-dependent fungicidal activity *in vitro* and a long half-life (∼133 h in humans, ∼70 to 80 h in mice). Given these characteristics, it is likely that the shape of the CD101 exposure (i.e., the time course of CD101 concentrations) influences efficacy. To test this hypothesis, doses which produce the same total area under the concentration-time curve (AUC) were administered to groups of neutropenic ICR mice infected with Candida albicans R303 using three different schedules. A total CD101 dose of 2 mg/kg was administered as a single intravenous (i.v.) dose or in equal divided doses of either 1 mg/kg twice weekly or 0.29 mg/kg/day over 7 days. The studies were performed using a murine disseminated candidiasis model. Animals were euthanized at 168 h following the start of treatment. Fungi grew well in the no-treatment control group and showed variable changes in fungal density in the treatment groups. When the CD101 AUC from 0 to 168 h (AUC_0–168_) was administered as a single dose, a >2 log_10_ CFU reduction from the baseline at 168 h was observed. When twice-weekly and daily regimens with similar AUC values were administered, net fungal stasis and a >1 log_10_ CFU increase from the baseline were observed, respectively. These data support the hypothesis that the shape of the CD101 AUC influences efficacy. Thus, CD101 administered once per week demonstrated a greater degree of fungal killing than the same dose divided into twice-weekly or daily regimens.

## INTRODUCTION

The echinocandin CD101 is a novel antifungal agent with activity against Aspergillus and Candida species, including azole- and echinocandin-resistant isolates (1, 2). This compound is a structural analog of anidulafungin but differs in certain beneficial ways with regard to toxicological and pharmacokinetic properties. Regarding the former, no changes were observed in the hepatocytes of Sprague-Dawdley rats exposed to supratherapeutic doses of CD101 in a 2-week repeated-dose study (3). In contrast, rats in this study administered comparable doses of anidulafungin displayed hepatocellular necrosis ranging from mild to moderate. Compared to anidulafungin and other echinocandins, CD101 has a considerably longer half-life (4–8). This finding was observed preclinically across multiple animal species (4) and in healthy volunteers (5). Results from the latter study demonstrated that the terminal half-life of CD101 is approximately 133 h (5), a value far greater than those reported for anidulafungin, caspofungin, and micafungin (9 to 52 h) (6–8).

CD101 demonstrates a concentration-dependent pattern of fungal killing (9), as previously observed with anidulafungin, caspofungin, and micafungin (10). This property, in conjunction with CD101's long half-life in humans, led to the hypothesis that a front-loaded CD101 dosing regimen would provide fungal killing superior to that of multiple-dose regimens. In order to test this hypothesis, front-loaded dose studies were conducted using a neutropenic murine disseminated candidiasis model. Our objective was to evaluate a single-dose regimen fractionated to yield multiple dosing regimens with equivalent areas under the concentration-time curve (AUC) for CD101 and to assess their corresponding changes in log_10_ CFU counts at 168 h to determine if exposure shape impacts antifungal activity.

## RESULTS

### *In vitro* susceptibility testing.

The modal CD101 MIC value against C. albicans R303, determined using broth microdilution, was 0.125 mg/liter.

### Pharmacokinetic study.

**FIG 1:**
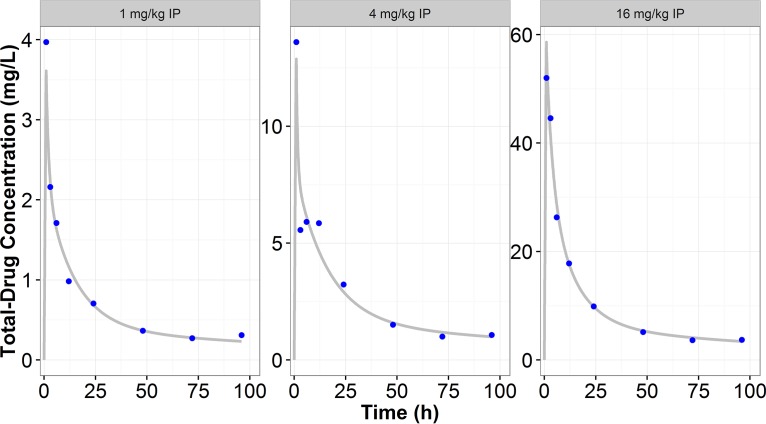
Observed (points) and model-predicted (lines) CD101 total-drug concentration (Conc.) versus time following i.p. administration of CD101.

Following intraperitoneal (i.p.) administration of CD101, maximum plasma concentrations were observed at the first sampling occasion (1 h) with values of 3.97, 13.6, and 52.0 mg/liter for the doses of 1, 4, and 16 mg/kg of body weight, respectively. CD101 exhibited linear pharmacokinetics over the studied dose range (1 to 16 mg/kg i.p.). A four-compartment model best described the disposition of this agent in murine plasma. Observed and model predicted pharmacokinetic profiles are displayed in Fig. 1. The coefficient of determination (*r*^2^) for the model-predicted concentrations versus observed concentrations was 0.97. Final model parameter estimates are displayed in Table 1. All model parameters were estimated with excellent precision, as indicated by a standard error of the mean (SEM) of less than 20% for 7 of 8 of the model parameters. Results of the protein binding evaluation demonstrated that the magnitude of CD101 protein binding in mouse serum was 99.2% across concentrations ranging from 7 to 60 mg/liter.

**TABLE 1:** Final CD101 pharmacokinetic model parameter estimates

Parameter^*a*^	Value	
	Final estimate	% SEM
CLt (liters/h/kg)	0.0104	3.55
V1 (liters/kg)	0.201	0.585
Q2 (liters/h/kg)	0.0153	1.24
V2 (liters/kg)	0.871	1.62
Q3 (liters/h/kg)	0.312	19.7
V3 (liters/kg)	0.0341	19.4
Q4 (liters/h/kg)	0.0723	43.8
V4 (liters/kg)	0.165	18.5

a CLt, total clearance; V1, central compartment volume; Q2, Q3, Q4 = distributional clearances; V2, V3, V4 = peripheral compartment volumes.

### Front-loaded dose studies.

**FIG 2:**
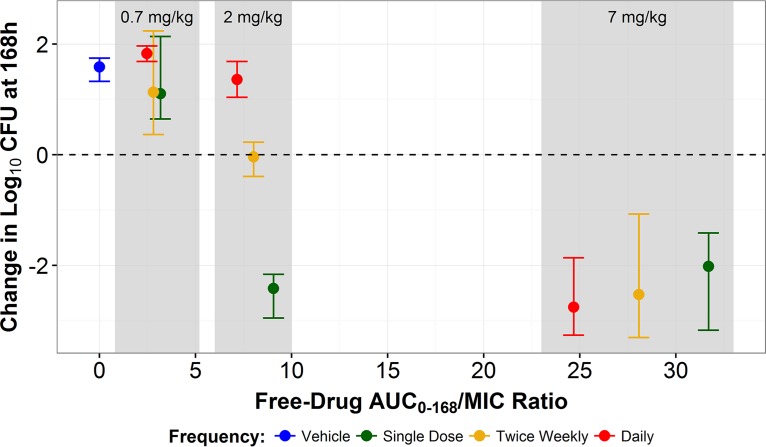
Mean change (solid circles) and range of change (error bars) in log_10_ CFU from the baseline at 168 h versus CD101 free-drug AUC_0−168_/MIC ratio by fractionation schedule.

**FIG 3:**
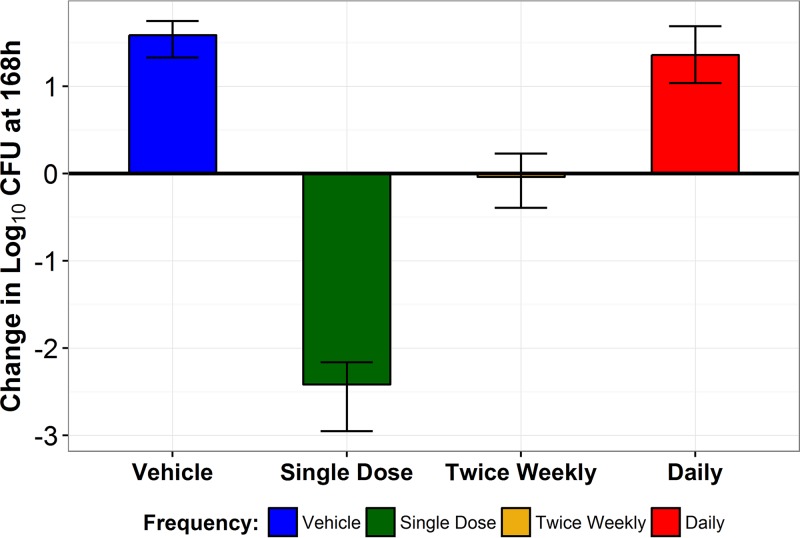
Mean change (bar) and range of change (error bars) in log_10_ CFU from the baseline at 168 h after administration of CD101 (2 mg/kg) by fractionation schedule.

The results of the front-loaded dose studies are presented in Fig. 2. Fungi in the no-treatment control group grew well and reached a density of greater than 1 × 10^6^ CFU/g by 48 h. The magnitudes of net change in log_10_ CFU from the baseline at 168 h were similar regardless of the fractionation schedule within the CD101 0.7 mg/kg and 7 mg/kg dosing groups. In the 0.7 mg/kg dose group, the magnitude of net change was similar to that measured for the no-treatment control group, regardless of the fractionation schedule. In the 7 mg/kg dose group, the magnitude of net change was greater than 2 log_10_ CFU reduction from the baseline at 168 h, regardless of the fractionation schedule. Conversely, results within the CD101 2 mg/kg group differed across the fractionation schedules. Those data are displayed in Fig. 3. When a total dose of 2 mg/kg was delivered daily (0.29 mg/kg/day), the magnitude of net change in log_10_ CFU from the baseline at 168 h was similar to the no-treatment control group result. However, when this dose was front-loaded (i.e., delivered entirely on day 1), there was a greater than 2 log_10_ CFU reduction from the baseline at 168 h. While these regimens, 0.29 mg/kg daily × 7 and 2 mg/kg × 1, resulted in comparable cumulative CD101 exposure at 168 h (Fig. 2), the treatment effects differed greatly.

**FIG 4:**
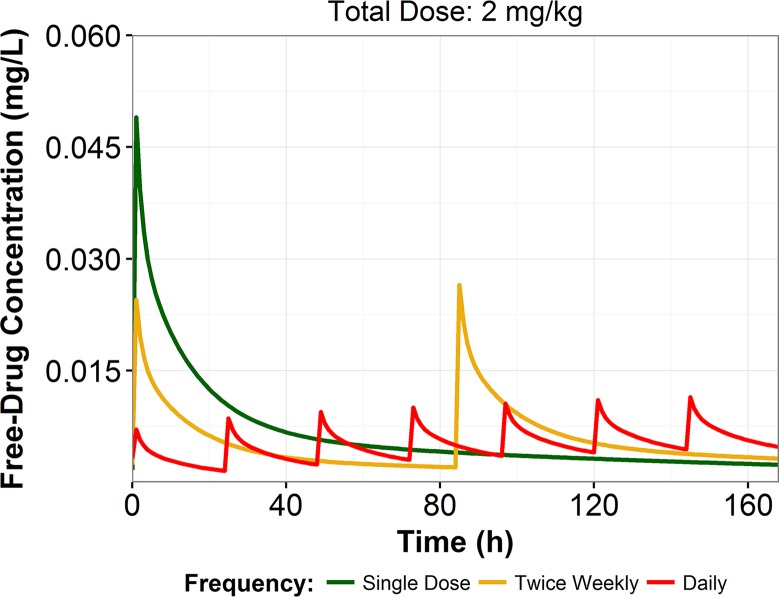
Simulated free-drug concentration-time profiles for the fractionated CD101 2-mg/kg regimens.

Simulated free-drug plasma concentration-time profiles of the three fractionated CD101 2 mg/kg dosing regimens are shown in Fig. 4. The regimens displayed dissimilar exposure profiles. In particular, the single-dose regimen resulted in substantially higher levels of CD101 exposure early in therapy. The free-drug plasma AUC values during the first 24 h of therapy (AUC_0–24_) were 0.520, 0.260, and 0.0754 mg·h/liter following administration of CD101 at 2 mg/kg as a front-loaded, twice-weekly, and daily regimen, respectively. Further, as shown in Fig. 4, administration of a front-loaded regimen resulted in free-drug plasma concentrations that remained above those measured for the twice-weekly and daily regimens for 84 and 48 h, respectively. No overt signs of toxicities were observed in any of the mice evaluated.

## DISCUSSION

The goal of these studies was to examine the impact of the shape of the drug exposure, as measured by AUC, on the fungicidal activity of CD101. Using a front-loading experiment design, in which similar AUC values were achieved through single, twice weekly, or daily dosing, we successfully demonstrated that the shape of the CD101 exposure greatly influenced fungicidal activity.

More specifically, a 2-log reduction in log_10_ CFU from the baseline was achieved for all five mice administered CD101 at 2 mg/kg of body weight (free-drug AUC_0–168_ = 1.13 mg·h/liter) as a single dose. However, when a twice-weekly regimen with a comparable AUC was administered, net stasis was observed. Further, when a regimen with a comparable AUC was administered daily over 7 days, a 1-log increase in log_10_ CFU from the baseline was observed (similarly to the no-treatment control group results). Given that echinocandins exhibit a concentration-dependent pattern of *in vitro* fungal killing (10) and that the front-loaded and daily CD101 regimens resulted in similar CD101 free-drug AUC_0–168_ values, one would expect to see similar magnitudes of fungal killing regardless of the fractionation schedule. However, the results of the front-loaded dose studies suggest that the shape of the CD101 AUC is a determinant of efficacy, with front-loaded regimens demonstrating greater benefit.

When CD101 was administered as a single dose, free-drug concentrations remained higher than those seen with the fractionated regimens over the first several days of therapy. CD101 free-drug plasma trough concentrations were comparable on day 5 for the front-loaded and daily dosing regimens. Additionally, the single-dose regimen yielded a free-drug CD101 AUC_0–24_ 7-fold greater than that yielded by the daily CD101 regimen. These pharmacokinetic characteristics provide support for the use of the front-loaded single-dose regimen, which is highly beneficial given the importance of achieving efficacious drug exposures early in therapy.

As demonstrated in Fig. 2, a daily 1 mg/kg dosing regimen was required to achieve a reduction in log_10_ CFU from the baseline over 168 h comparable to that obtained after administration of a front-loaded 2 mg/kg regimen. Administration of the daily 1 mg/kg regimen resulted in a CD101 AUC_0–168_ that was approximately 3-fold greater than that seen with the 2 mg/kg front-loaded regimen. Thus, front-loaded CD101 doses provided comparable levels of efficacy and reduced levels of total drug exposure, characteristics which may reduce the risk of AUC-driven drug-related toxicities.

Similar findings were seen during the development of oritavancin, a lipoglycopeptide antibacterial indicated for the treatment of patients with acute bacterial skin and skin structure infections due to Gram-negative organisms. Oritavancin is another agent which exhibits a remarkably long half-life (terminal half-life, ∼245 h) (11). This agent is unique in that a full course of therapy consists of a single 1,200-mg intravenous dose. Two characteristics of oritavancin's pharmacokinetics and pharmacodynamics enable this front-loaded dosing regimen to provide prolonged efficacious drug exposures and produce outcomes superior to those seen with multiple-dose regimens (12, 13). The first of these, a pharmacodynamic characteristic, is that oritavancin displays a concentration-dependent pattern of *in vitro* bacterial killing (14). Given this characteristic, as the drug concentration increases, so too do the rate and extent of bacterial killing. The second characteristic is oritavancin's distinct pharmacokinetic profile, which provides consistent exposure over a prolonged period. This is a product of the protracted terminal half-life of oritavancin. As stated previously, CD101 exhibits a concentration-dependent pattern of *in vitro* fungal killing and possesses a long half-life.

Several authors have demonstrated findings similar to those described here for other echinocandins with shorter plasma half-lives with respect to reductions in CFU associated with the administration of front-loaded dosing regimens that were similar to or greater than those seen with more-fractionated dosing regimens in mice (15–18). Those investigators postulated that their results were due to therapeutic drug concentrations being maintained within peripheral tissues over a prolonged period. However, in order to translate those findings to humans, two assumptions must be made: first, that the magnitude of echinocandin distribution in peripheral tissue in humans is similar to or greater than that in mice; second, that the time courses of echinocandin disposition into and out of peripheral tissues are similar in humans and mice. Using the examples of ceftobiprole and oritavancin for the former and later assumptions, respectively, these characteristics cannot always be assumed to be concordant across species (19). Additional studies characterizing the disposition of CD101 in tissue in both mice and humans may be useful to further interpret these data.

In summary, the studies described here demonstrated that front-loaded CD101 dosing regimens exhibited greater fungicidal activity than more-fractionated regimens. This apparent relationship between exposure shape and fungal killing greatly differentiates CD101 from the antifungals used in currently approved therapies and poses several beneficial clinical implications. Front-loaded dosing provides the opportunity to deliver drug exposures in a pharmacokinetic/pharmacodynamic-optimized manner, to improve patient compliance, and to reduce the resources required for therapeutic drug monitoring. These data for CD101 provide dose selection support for future clinical studies.

## MATERIALS AND METHODS

### Study drug, challenge isolate, and *in vitro* susceptibility testing.

CD101 was supplied by Cidara Therapeutics, Inc. (San Diego, CA). Stock solutions of CD101 were prepared in dimethyl sulfoxide (DMSO) for the susceptibility testing. The vehicle used for dosing in the *in vivo* studies was 10% DMSO–1% Tween 20–saline solution. The challenge isolate utilized in these studies was Candida albicans R303. Susceptibility testing was done in accordance with the Clinical and Laboratory Standards Institute (CLSI) M27-A3 methods, using RPMI 1640 medium, an initial inoculum of 0.5 × 10^3^ to 2.5 × 10^3^ CFU/ml, and incubation at 35°C (20). MIC plates were read following 24 h of incubation, and MIC values are reported as concentrations resulting in prominent (∼50%) growth inhibition. Susceptibility testing was performed on three separate occasions. Final results were expressed as modal values. Quality control was performed on each day of testing using CLSI-recommended reference strains (C. krusei ATCC 6258 and C. parapsilosis ATCC 22019).

### Pharmacokinetic study.

Healthy female ICR mice weighing 20 to 24 g were administered a single dose of CD101 via intraperitoneal (i.p.) injection. Doses of 1, 4, and 16 mg/kg of body weight were studied, utilizing 3 animals per dose level. Whole-blood samples (one sample per animal) were collected at 1, 3, 6, 12, 24, 48, 72, and 96 h postdose. Plasma was collected by centrifugation, and samples were stored at −20°C or below. Samples were analyzed using liquid chromatography with tandem mass spectrometry detection and an AB-SCIEX API 4000 mass spectrometry system. The method used in the analysis was qualified as fit for the purpose: calibration standards were matrix matched, and, for each analytical batch, triplicate calibration standards were included at the beginning of the batch, approximately in the middle, and at the end. Following analysis, quantitation is carried out by a calibration curve comprising analyte/internal standard area ratio versus concentration. Calibration standards ranged from 0.5 mg/liter (lower limit of quantitation [LLOQ]) to 100 mg/liter. Analytical batch acceptance followed the general guidance criterion that standards/quality control should be within ±20% for accuracy (percent nominal concentration) and ≤20% for precision (percent coefficient of variation). For analysis, plasma samples were quenched with acetonitrile (4:1 acetonitrile/plasma ratio) containing diclofenac as the internal standard. Animals were maintained in accordance with the criteria of the American Association for Assessment and Accreditation of Laboratory Animal Care (AAALAC). The pharmacokinetic study was approved by the Animal Research Committee of the William S. Middleton Memorial Veterans Affairs Hospital and the University of Wisconsin.

### Protein binding.

The extent of protein binding of CD101 to murine K_2_EDTA plasma proteins was determined using ultracentrifugation (500,000 × *g* for 2.5 h at 37°C). CD101 was tested at concentrations of 7, 10, 20, 30, and 60 mg/liter. An experimental control was included in order to assess compound recovery (matrix stability) under the assay conditions. The level of protein binding was calculated using the following equation: percent protein binding = 100% − percent unbound (where “percent unbound” represents mean supernatant concentration/total mean concentration × 100).

### Front-loaded dose studies.

A neutropenic, murine, disseminated candidiasis model was used for the evaluation of the effects of the CD101 dosing regimens on efficacy. Male or female ICR mice (*n* = 5 per regimen and observation time) weighing 22 ± 2 g were rendered neutropenic by treatments using the injection of cyclophosphamide 4 days prior to infection (day −4) and 1 day prior to infection (day −1) at 150 and 100 mg/kg i.p., respectively. Neutropenia was sustained for the duration of the study with cyclophosphamide doses (100 mg/kg i.p.) administered every 48 h on days 1, 3, 5, and 7 after infection. Each animal was inoculated, using a lateral tail vein injection, with 1 × 10^3^ CFU of Candida albicans (isolate R303). CD101 (or vehicle) was administered 24 h postinfection via i.p. injection. The dosing regimens studied are shown in Table 2. Mice were euthanized with CO_2_ at 168 h (7 days) following the start of treatment. Mice in the control group were euthanized 0, 24, and 48 h after administration of vehicle. Paired kidneys were aseptically harvested and homogenized, and serial dilutions were plated for colony counts to determine the fungal burden (measured in CFU per gram). Kidney counts were not determined in animals that expired prior to the scheduled sacrifice time. The front-loaded dose studies was performed under animal biosafety level 2 conditions in an Association for Assessment and Accreditation of Laboratory Animal Care-accredited vivarium, at Eurofins Panlabs Taiwan, Ltd., with the oversight of veterinarians to ensure compliance with the Eurofins Panlabs Institutional Animal Care and Use Committee regulations and the humane treatment of laboratory animals.

**TABLE 2:** Summary of CD101 dosing regimens evaluated in front-loaded dose studies

Total dose	Dosing schedule^*a*^	Fractionated dose(s)
0.7 mg/kg i.p.	Single	0.7 mg/kg × 1
	Twice weekly	0.35 mg/kg × 2
	Daily	0.1 mg/kg × 7
2 mg/kg i.p.	Single	2 mg/kg × 1
	Twice weekly	1 mg/kg × 2
	Daily	0.29 mg/kg × 7
7 mg/kg i.p.	Single	7 mg/kg × 1
	Twice weekly	3.5 mg/kg × 2
	Daily	1 mg/kg × 7

a For the twice-weekly dosing schedule, the second dose was administered 84 h (3.5 days) after first dose.

### Data analysis.

Data collected from the pharmacokinetic study described above were used to develop a pharmacokinetic model describing the disposition of CD101 in mice in S-ADAPT (21). The developed pharmacokinetic model, along with the unbound CD101 fraction, was used to simulate free-drug concentration-time profiles for each dosing regimen administered in the front-loaded dose studies. Free-drug AUC_0–168_ values were calculated through numeric integration of CD101 free-drug concentration-time profiles. Relationships between changes in log_10_ CFU at 168 h and free-drug AUC_0–168_/MIC ratios were explored.
